# Bipiperidinyl Derivatives of Cannabidiol Enhance Its Antiproliferative Effects in Melanoma Cells

**DOI:** 10.3390/antiox13040478

**Published:** 2024-04-17

**Authors:** Peihong Lyu, Huifang Li, Junzhao Wan, Ying Chen, Zhen Zhang, Panpan Wu, Yinsheng Wan, Navindra P. Seeram, Jean Christopher Chamcheu, Chang Liu, Hang Ma

**Affiliations:** 1 Bioactive Botanical Research Laboratory, Department of Biomedical and Pharmaceutical Sciences, College of Pharmacy, University of Rhode Island, Kingston, RI 02881, USA; peihong.lyu@uri.edu (P.L.); huifang_li@uri.edu (H.L.);; 2 Department of Dermatology, Affiliated Hospital of Guizhou Medical University, Guiyang 550001, China; 3 School of Pharmacy, Guizhou Medical University, Guiyang 550001, China; 4 Department of Obstetrics and Gynecology, The Second Affiliated Hospital of Soochow University, Suzhou 215004, China; 5 Guangdong Provincial Key Laboratory of Large Animal Models for Biomedicine, School of Pharmacy and Food Engineering, Wuyi University, Jiangmen 529020, China; 6 Department of Biology, Providence College, Providence, RI 02918, USA; 7 Department of Biological Sciences and Chemistry, College of Sciences and Engineering, Southern University and A&M College, Baton Rouge, LA 70813, USA; 8 Department of Pathobiological Sciences, School of Veterinary Medicine, Louisiana State University, Baton Rouge, LA 70803, USA

**Keywords:** cannabinoids, cannabidiol, bipiperidinyl, skin cancer, melanoma, apoptosis, ferroptosis, apoptosis

## Abstract

Cannabis and its major cannabinoid cannabidiol (CBD) are reported to exhibit anticancer activity against skin tumors. However, the cytotoxic effects of other minor cannabinoids and synthetic CBD derivatives in melanoma are not fully elucidated. Herein, the antiproliferative activity of a panel of phytocannabinoids was screened against murine (B16F10) and human (A375) melanoma cells. CBD was the most cytotoxic natural cannabinoid with respective IC_50_ of 28.6 and 51.6 μM. Further assessment of the cytotoxicity of synthetic CBD derivatives in B16F10 cells identified two bipiperidinyl group-bearing derivatives (**22** and **34**) with enhanced cytotoxicity (IC_50_ = 3.1 and 8.5 μM, respectively). Furthermore, several cell death assays including flow cytometric (for apoptosis and ferroptosis) and lactate dehydrogenase (for pyroptosis) assays were used to characterize the antiproliferative activity of CBD and its bipiperidinyl derivatives. The augmented cytotoxicity of **22** and **34** in B16F10 cells was attributed to their capacity to promote apoptosis (as evidenced by increased apoptotic population). Taken together, this study supports the notion that CBD and its derivatives are promising lead compounds for cannabinoid-based interventions for melanoma management.

## 1. Introduction

Skin cancers, including basal cell carcinoma, squamous cell carcinoma, and melanoma, present a significant global health challenge due to their widespread prevalence and the associated high morbidity and mortality rates [1]. Among these, melanoma, though less prevalent than basal cell and squamous cell carcinomas, is particularly notorious for its aggressive nature and propensity for rapid metastasis. Treatment strategies for melanoma, adjusted according to the tumor’s stage and location, often involve surgery, immunotherapy, and chemotherapy [2]. In instances where first-line treatments like immunotherapy are unsuitable, chemotherapy drugs are employed. Notably, natural product-derived anticancer agents, including paclitaxel and vinblastine, are commonly used in conjunction with other therapies to mitigate drug resistance [3].

A considerable amount of research effort has been directed at discovering natural products (and their derivatives) with anti-melanoma effects. Preclinical studies demonstrated that cannabis and its bioactive compounds, namely, cannabinoids, present promising effects on the inhibition of tumor growth and reduction in tumor size [4]. For instance, it was reported that cannabidiol (CBD), a major non-psychoactive cannabinoid in cannabis, can decrease the growth rate of melanoma tumors and increase the animal survival curve in a murine melanoma model [5]. However, the underlying mechanisms of CBD’s anti-melanoma effects remain to be fully elucidated.

Our group has initiated a program to systematically evaluate CBD’s multifaceted biological activities including anti-inflammation, antioxidant, and inhibitory effects on enzymes (including cholinesterases, kynurenine-3-monooxygenase, and virus main protease) [6,7,8,9]. Additionally, we have explored CBD’s antimicrobial effects against methicillin-resistant *Staphylococcus aureus* [10]. Furthermore, a panel of CBD’s derivatives was chemically synthesized for augmented antibacterial activities, which provided insights into the structural–activity relationship (SAR) for CBD’s mechanism of action.

However, the challenges remain as to whether the antiproliferative effect of CBD on melanoma cells can be enhanced by structural modifications. The objectives of this study are (1) to evaluate the antiproliferative effects of phytocannabinoids, including CBD, against both murine (B16F10) and human (A375) melanoma cell lines; (2) to investigate how structural modifications to CBD influence its antiproliferative effects; and (3) to explore the effects of CBD and its derivatives on various forms of programmed cell death including apoptosis, ferroptosis, and pyroptosis.

## 2. Materials and Methods

### 2.1. Chemical and Reagents

**Figure 1:**
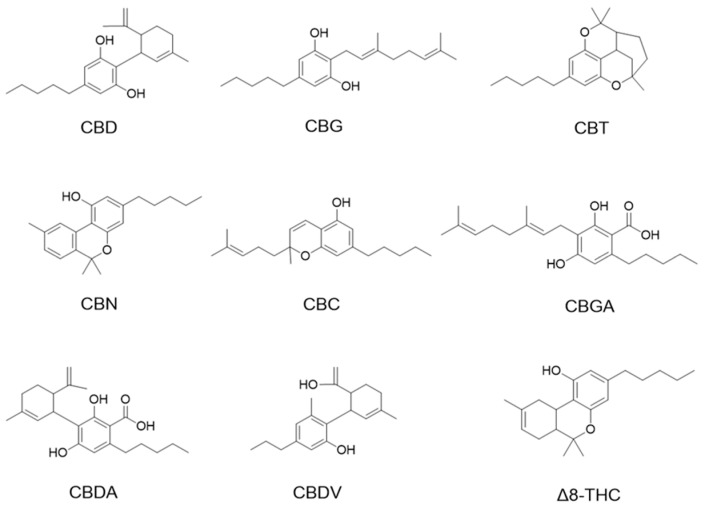
Chemical structures of phytocannabinoids were evaluated in this study.

Cannabinoids including cannabidiol (CBD), cannabigerol (CBG), cannabicitran (CBT), cannabinol (CBN), cannabichromene (CBC), cannabigerolic acid (CBGA), cannabidiolic acid (CBDA), and cannabidivarin (CBDV) delta-8-tetrahydrocannabinol (Δ8-THC) were purchased from Cayman Chemical (Ann Arbor, MI, USA; see their chemical structures in Figure 1).

Chemicals including the erastin, etoposide, and lactate dehydrogenase (LDH) assay kit were purchased from Cayman Chemicals. The derivatives of CBD (**1**–**56**) were chemically synthesized by our laboratory with a protocol previously reported [10]. Phosphate-buffered saline (PBS), dimethyl sulfoxide (DMSO), 3-(4,5-dimethylthiazol-2-yl)-2,5-diphenyltetrazolium bromide (MTT), 2′,7′-dichlorofluorescin diacetate fluorescent probe (DCF-DA), and crystal violet were purchased from Sigma-Aldrich, Co. (St. Louis, MO, USA). Paraformaldehyde and the Alexa Fluor^TM^ 488 Annexin V/Dead cell apoptosis kit were purchased from Thermo Fisher Scientific (Waltham, MA, USA). The Liperfluo and FerroOrange assay kits were obtained from Dojindo Lab (Kumamoto, Japan).

### 2.2. Cell Culture and Viability Assay

Murine melanoma (B16F10) cells and human melanoma (A375) cells were purchased from the American Type Culture Collection (ATCC; Rockville, MD, USA) and cultured according to protocols by ATCC. The Dulbecco’s modified Eagle’s medium (DMEM; Life Technologies, Gaithersburg, MD, USA) supplemented with 10% fetal bovine serum (Life Technologies) and 1% antibiotic solution (Sigma-Aldrich, Co., St. Louis, MO, USA) was used for the culture. The cell viability was measured by the MTT assay [11]. Briefly, cells were maintained at 37 °C in a 5% CO_2_ incubator, which has a 95% relative humidified atmosphere. Test samples were dissolved in DMSO as a stock solution (100 mM) and then diluted with the cell culture medium to the desired concentrations. The final DMSO concentration was <0.1%. Cells were seeded in 96-well plates at 5 × 10^3^ cells/mL in 100 μL of cultural medium in quadruplicate and cultured overnight. Then, the culture medium was replaced with fresh medium containing test samples at various concentrations (1–100 μΜ). After treatment with test samples (24 h), the MTT reagent (20 μL) was added and incubated for 4 h. The absorbance of each well at 570 nm was recorded with a SpectraMax M2 plate reader (Molecular Devices, Sunnyvale, CA, USA). The inhibition of proliferation by the sample treatment is expressed as a percentage compared to the control cells (with ≤0.1% DMSO). Data are presented as mean values ± standard error of the mean (SEM) and were obtained from four separate experiments.

### 2.3. Cell Colony Formation Assay

B16F10 cells were seeded in 6-well plates at a density of 1 × 10^3^ cells/well and allowed to grow for 24 h. CBD (40 μM) and its derivatives **22** (10 μM) and **34** (10 μM), which are bipiperidinyl moiety-containing analogs with the most promising antiproliferative effect selected from a panel of synthetic cannabinoids, were included for the cell colony assay. After treatment of the test samples for 12 h, the culture medium was replaced with a fresh medium for 12 h. The culture medium was then changed every three days. After 7 days, cells were washed with ice-cold PBS and the clones were fixed with 4% paraformaldehyde for 20 min and then stained with 0.1% crystal violet for 15 min. Subsequently, the excess crystal violet solution was removed by slowly washing the cells with PBS. Colonies were photographed and quantified. The colony numbers were counted using the software of Image J Version 1.52 (http://rsb.info.nih.gov/ij/; accessed on 19 October 2023).

### 2.4. Detection of Apoptosis and Necrosis

Apoptosis and necrosis cells were assessed by flow cytometric assay [11]. Briefly, B16F10 cells were cultured at a density of 2 × 10^5^ in 6-well plates for 12 h and then treated with CBD, **22**, and **34** at concentrations that were near their IC_50_ of the cytotoxicity for 24 h. Next, B16F10 cells were collected and labeled with FITC-conjugated-annexin V and PI reagents according to the manufacturer’s instructions. Then, the population of apoptotic and necrotic cells was measured by flow cytometry (BD FACSCalibur, San Jose, CA, USA) and data were analyzed using the software FlowJo v8 (LLC, Ashland, OR, USA).

### 2.5. Measurement of Ferroptosis

The effects of CBD and its derivatives on ferroptosis were evaluated with two methods including lipid peroxidation and cellular ion assays. The content of lipid peroxide (LPO) was detected by the Liperfluo assay [12]. Briefly, B16F10 cells were cultured at a density of 2 × 10^5^ in 6-well plates overnight and then CBD (40 and 50 μΜ), **22** (5 and 10 μΜ), and **34** (10 and 15 μΜ) were added after cell adhesion. Next, cells were collected and washed with PBS and then labeled with Liperfluo reagent in the incubator for 30 min. Then, cells were washed with PBS twice. The fluorescence signals were detected and analyzed by flow cytometer (BD FACSCalibur) and data were analyzed using the software FlowJo. The content of intracellular ion (Fe^2+^) was detected by the FerroOrange staining assay [13]. Briefly, B16F10 cells (2 × 10^5^ cells per well) were seeded in a 6-well plate and incubated overnight for adherence. Cells were collected after 24 h of treatment with CBD, **22**, and **34** and then washed with PBS and incubated with the FerroOrange reagent in the dark at 37 °C for 30 min. Finally, they were assayed by flow cytometry and analyzed by the software FlowJo, which is the same as the above method.

### 2.6. Detection of Reactive Oxygen Species

The cellular oxidative stress was measured by the detection of reactive oxygen species [14]. Briefly, B16F10 cells were cultured at a density of 2 × 10^5^ in 6-well plates and treated with CBD (40 μM), **22** (5 μM), and **34** (10 μM) for 24 h. Then, cells were collected and treated with DCF-DA (20 μM) for 30 min before being washed with PBS twice. Then, cells were quantified by flow cytometry and analyzed by the software FlowJo.

### 2.7. Detection of LDH

The lactate dehydrogenase levels of cells treated with test samples were evaluated by an LDH kit according to the manufacturer’s instructions [7]. Briefly, B16F10 cells were cultured at a density of 5 × 10^3^ in a 96-well plate and treated with CBD, **22**, and **34** (40 μM) for 24 h. Then, the supernatant was transferred to a 96-well plate followed by adding the LDH reaction solution with gentle shaking for 30 min at 37 °C. The absorbance of each well at a wavelength of 490 nm was recorded with a SpectraMax M2 plate reader.

### 2.8. Statistical Analysis

Statistical analyses were performed using GraphPad Prism 10 (GraphPad Software, La Jolla, CA, USA). Data are expressed as the mean value ± standard deviation (SD) obtained from triplicates of experiments. The significance of differences was determined using a one-way analysis of variance (ANOVA).

## 3. Results and Discussion

### 3.1. Phytocannabinoids Inhibit the Proliferation of Murine and Human Melanoma Cells

We first evaluated the antiproliferative effects of natural phytocannabinoids (including CBD, CBG, CBT, CBN, CBC, CBGA, CBDA, CBDV, and Δ8-THC; chemical structures are shown in Figure 1) in murine (B16F10) and human (A375) melanoma cells. At a lower concentration (10 μM), these phytocannabinoids showed weak antiproliferative effects with up to 27.2% inhibition (Table 1). Their antiproliferative effects were stronger at 100 μM and several phytocannabinoids including CBD, CBN, CBG, CBDV, and Δ8-THC inhibited the growth of B16F10 and A375 cells by 84.7–93.7% and 82.7–94.7%, respectively. Etoposide was used as a positive control and it showed antiproliferative effects by 46.7% and 78.2% inhibition at 10 μM in B16F10 and A375 cells, respectively.

**Figure 2:**
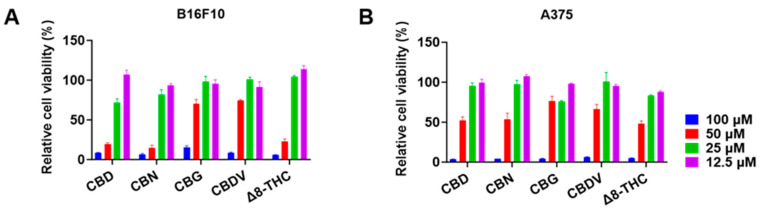
Effects of phytocannabinoids including CBD, CBN, CBG, CBDV, and Δ8-THC on the proliferation of melanoma cells including B16F10 (**A**) and A375 (**B**) cells for 24 h.

The antiproliferative effects of CBD, CBN, CBG, CBDV, and Δ8-THC in B16F10 and A375 cells were further evaluated at various concentrations (12.5, 25, 50, and 100 μM) for 24 h. CBD, CBN, and Δ8-THC at 25 and 50 μM showed an inhibition of 28.0% and 80.2%, 18.0% and 85.5%, and −4.3% and 77.0%, respectively, in B16F10 cells (Figure 2A). A similar trend was observed where these cannabinoids (25 and 50 μM) inhibited the growth of A375 cells by 4.7% and 48.0%, 2.5% and 46.3%, and 16.5% and 51.7%, respectively (Figure 2B). The antiproliferation IC_50_ values of CBD, CBN, CBG, CBDV, and Δ8-THC are shown in Table 2.

**Figure 3:**
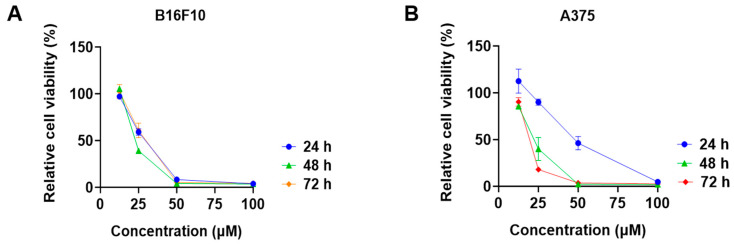
Effects of CBD on the viability of melanoma cell lines including B16F10 (**A**) and A375 (**B**) at time points of 24 h, 48 h, and 72 h.

Among the tested phytocannabinoids, CBD had the most promising antiproliferative effects in B16F10 cells with an IC_50_ of 28.6 μM. We further evaluated the cytotoxicity of CBD in melanoma cells at multiple time points. CBD had comparable inhibitions on the growth of B16F10 cells at the time points of 24, 48, and 72 h (Figure 3A), whilst its antiproliferative effects were more significant for the longer treatment times (24 and 72 h) in A375 cells (Figure 3B).

Although several cannabis extracts have been reported to exert inhibitory effects on the growth of melanoma cells [15,16], only limited studies explored the antiproliferative activity of the individual cannabinoids. A study reported that CBD and CBG showed the most promising antiproliferative effect (IC_50_ = 12.0 and 12.1 μM, respectively) compared to other minor cannabinoids, such as CBN and CBC, in human melanoma A375 cells [17]. It was also reported that CBD had the highest inhibitory effect on the growth of murine melanoma cells (B16F10) with an IC_50_ of 80 μM compared to other minor cannabinoids [17]. This trend was in agreement with data from our current study showing that the cytotoxicity of CBD was higher than other minor cannabinoids in murine melanoma cells. Next, we sought the augmented antiproliferative effect in B16F10 cells by modifying CBD’s structure.

### 3.2. Effect of CBD Derivatives on the Growth of Murine Melanoma Cells

**Figure 4:**
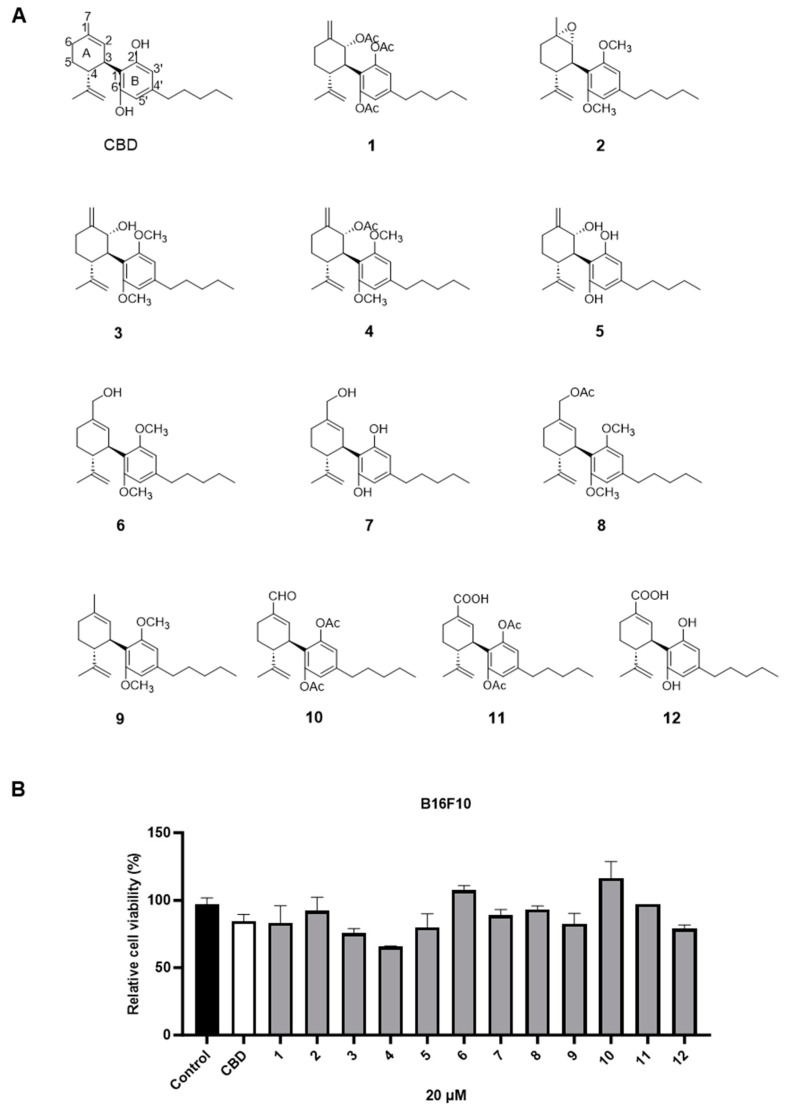
Chemical structures of CBD synthetic analogs **1**–**12** (**A**) and their effects on the cell viability of B16F10 melanoma cells at 20 µM (**B**).

The cytotoxic effects of a panel of synthetic CBD derivatives (**1**–**56**) in B16F10 cells were evaluated. In the primary screening, 12 CBD derivatives were synthesized with structural modifications including oxidation at the A ring of CBD (**1**–**5**) and various group substitutions at the 7-, 2′-, and 6′-positions (**6**–**12**) (Figure 4A). The antiproliferative effects of these synthetic analogs (at 20 µM) in B16F10 were assessed. Several compounds including **3**, **4**, **5**, and **12** showed improved cytotoxicity compared to CBD by 10.7%, 22.5%, 5.3%, and 6.5%, respectively (Figure 4B).

As CBD derivatives (**1***–***12**) showed limited improvement in the antiproliferative effects in B16F10 cells, we made additional structural modifications by introducing various functional groups in CBD at position 7 and in CBD analogs with two acetoxy groups (-OAc) replacing the phenol groups in the B ring. In the secondary screening, several CBD analogs showed enhanced antiproliferative effects in B16F10 and A375 cells. For instance, compounds **15** (-carboxamide), **18** and **30** (-methylpiperazine), **19** (-phenylpiperazine), **22** and **34** (-bipiperidine), **23** (-oxycyclohexane), and **36** (-chlorophenyl) showed a higher inhibition (41.7%, 46.5% and 66.2%, 48.0%, 86.5% and 88.5%, 66.5%, and 32.2%, respectively) compared to CBD (Table 3). A similar trend was observed as compounds **15**, **22**, **34**, and **36** showed enhanced antiproliferative effects in A375 cells.

### 3.3. Bipiperidinyl Derivatives Enhance the Antiproliferative Effect of CBD

**Figure 5:**
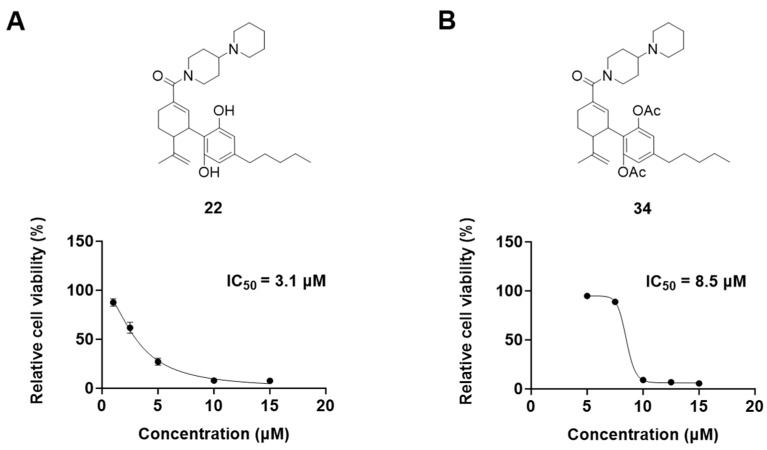
Chemical structure and effects on the viability of compound **22** (**A**) and compound **34** (**B**) in B16F10 cells at multiple concentrations.

It was noted that the CBD bipiperidinyl derivatives including **22** and **34** showed superior inhibitory effects on the growth of both B16F10 and A375 cells in the secondary screening. To confirm the antiproliferative effects of these two lead compounds, we further evaluated their cytotoxicity at multiple concentrations in B16F10 cells. Compound **22** was assayed at concentrations of 1, 2.5, 5, 10, and 15 μM, which showed an inhibition of 12.5%, 38.3%, 73.0%, 92.2%, and 92.5%, respectively, on the cell growth. Compound **34** was assayed at concentrations of 5, 7.5, 10, 12.5, and 15 μM and it showed antiproliferations of 5.3%, 11.3%, 91.0%, 93.2%, and 94.5%, respectively. The antiproliferation IC_50_ of **22** and **34** was 3.1 μM and 8.5 μM, respectively (Figure 5).

**Figure 6:**
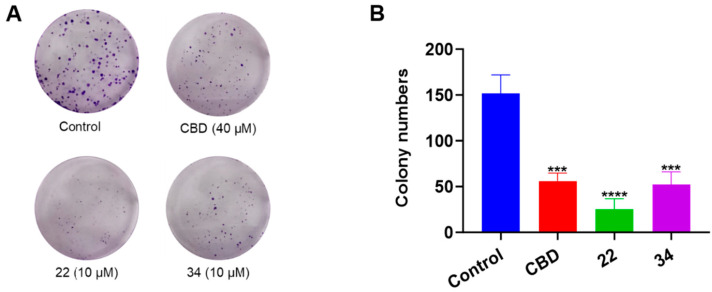
Effects of CBD, **22,** and **34** on the formation of B16F10 cell colonies measured by a crystal violet staining assay (**A**) and the quantitative analysis of colony numbers treated with CBD (40 µM), **22** (10 µM), and **34** (10 µM). (**B**) Values are expressed in means ± SD from three experiment replicates. Significance was defined as *** *p* < 0.001 and **** *p* < 0.0001 when compared to the control group.

Additionally, the antiproliferative effects of CBD, **22**, and **34** were compared in a colony formation assay. Treatment with CBD (40 μM) showed a moderate reduction in colony formation compared to the vehicle control group, whilst compounds **22** and **34** (both at 10 μM) significantly reduced colony formation by 83.5% and 65.6%, respectively (Figure 6).

The reaction intermediates **37**–**49** with leaving group (i.e., triflate; -OTf) and protecting group (pivaloyl; Piv) were inactive in the antiproliferative assay (Supplementary Materials Table S1). In addition, a group of CBD derivatives (**50**–**56**) with various side chains in the B-ring showed drastic activities. Compounds with a cyclohexane (**54**) and a cyclopentane (**55**) side chain showed inhibition of 55.2% and 61.7%, respectively, whereas the other derivatives were inactive (Supplementary Materials Table S2).

The SAR analysis suggested that the introductions of nitrogen-containing rings to CBD, i.e., piperazine (**18** and **30**) and piperidine group (**22** and **34**), can lead to the augmentation of cytotoxicity in B16F10 cells. This was not surprising as these functional groups are common moieties in several clinically used drugs. For instance, piperacillin, a broad-spectrum β-lactam antibiotic, has a bipiperidine side chain that enhances its penetration into Gram-negative bacteria [18]. In addition, it is reported that bipiperidine-containing agents such as DNA-intercalating polyketide glycosides can exert enhanced antiproliferative effects against human leukemia cells and human cervix carcinoma cells [19]. It was suggested that the enhanced antiproliferative effects of these bipiperidine sugar surrogates were partially due to their improved water solubility. However, further mechanistic studies are warranted to confirm that the augmented antiproliferative effect of CBD bipiperidine derivatives was attributed to their solubility. Moreover, given that the mediation of programmed cell death is a critical mechanism for anti-cancer agents with piperidine moiety [20], we next evaluated the effects of CBD and its bipiperidine derivatives on various types of programmed cell death in B16F10 cells.

### 3.4. CBD Bipiperidinyl Derivatives Induce Apoptosis in B16F10 Cells

**Figure 7:**
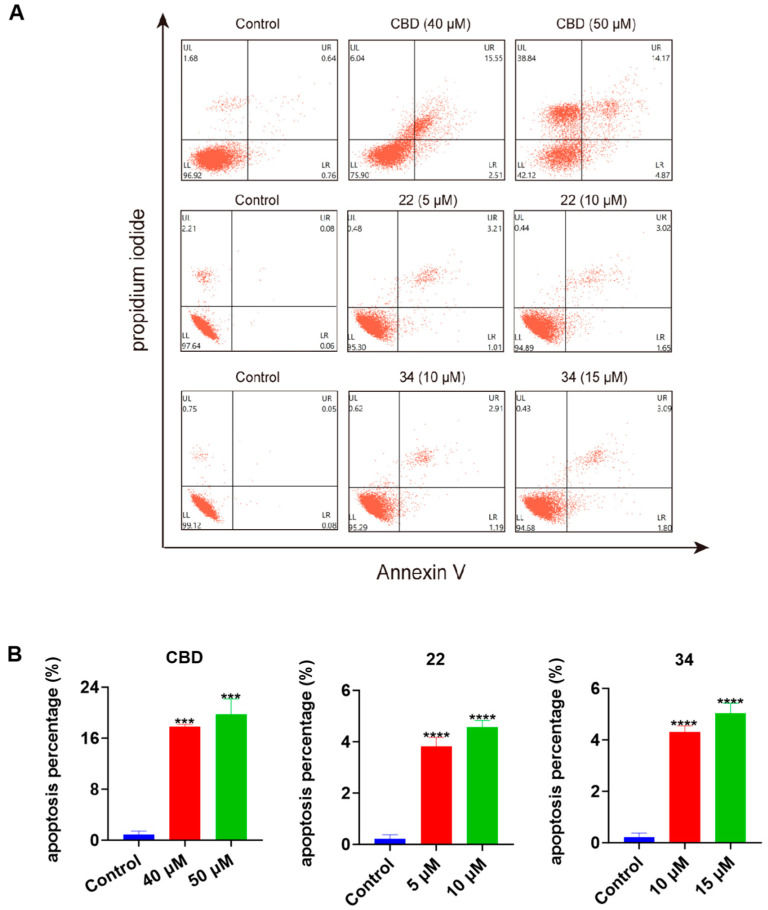
Effects of CBD, **22**, and **34** on the population of apoptotic B16F10 cells. Flow cytometry plots of B16F10 cells exposed to CBD (40 and 50 μM), **22** (5 and 10 μM), and **34** (10 and 15 μM) induced apoptosis (**A**). Changes in the percentage of the apoptosis index of cells exposed to CBD, **22**, and **34** (**B**). Values are expressed in means ± SD from three experiment replicates. Significance was defined as *** *p* < 0.001 and **** *p* < 0.0001 when compared to the control group.

To further explore the possible mechanisms of the lead compounds’ antiproliferative activity, the effects of CBD and its derivatives **22** and **34** on various types of programmed cell death including apoptosis, ferroptosis, and pyroptosis were assessed. In a flow cytometric assay, the induction of apoptosis by CBD, **22**, and **34** in B16F10 cells was detected with Annexin V/PI double staining reagent. Compared to the control group, CBD (40 and 50 μM), **22** (5 and 10 μM), and **34** (10 and 15 μM) increased the population of cells with late apoptosis (Figure 7A). Quantitative analysis of the apoptosis index showed that CBD (50 μM), **22** (10 μM), and **34** (15 μM) increased the overall population of apoptotic cells by 18.9%, 4.4%, and 4.8%, respectively (Figure 7B).

### 3.5. CBD Bipiperidinyl Derivatives Promote Lipid Peroxidation in B16F10 Cells

**Figure 8:**
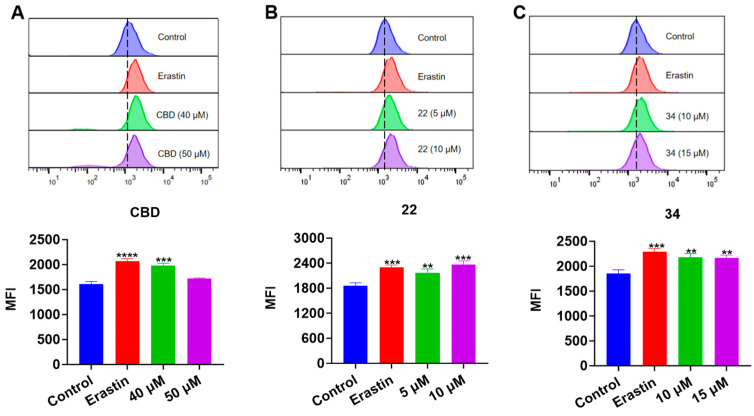
Evaluations of lipid peroxidation in B16F10 cells exposed to erastin, CBD, **22**, and **34**. Plots of a flow cytometry assay with the Liperfluo staining reagent showing the lipid peroxide levels (upper panel; data are shown as the FITC values) and quantitative analysis of the mean fluorescence intensity (lower panel; data are expressed as the mean ± SD MFI values) in B16F10 cells exposed to CBD, **22**, and **34** (**A**–**C**). Significance was defined as ** *p* < 0.01, *** *p* < 0.001, and **** *p* < 0.0001 when compared to the control group.

Next, we evaluated the effects of CBD and its derivatives on ferroptosis, which is a form of programmed cell death mediated by cellular lipid peroxidation and iron levels. Lipid peroxidation in B16F10 cells exposed to erastin (a ferroptosis inducer as the positive control), CBD, **22**, and **34** were detected by the Liperfluo assay. It showed that the cellular lipid peroxidation in cells exposed to CBD (40 μM), **22** (5 and 10 μM), and **34** (10 and 15 μM) elevated by 22.8%, 17.2% and 27.3%, and 17.4% and 16.8%, respectively (Figure 8). Erastin (15 μΜ), a known ferroptosis inducer, increased the cellular lipid peroxidation by 23.8%. These findings suggest that CBD and its derivatives **22** and **34** may promote ferroptosis in B16F10 cells but further investigation of ferroptosis-related biomarkers (i.e., the cellular iron level) is warranted to confirm the occurrence of ferroptosis.

**Figure 9:**
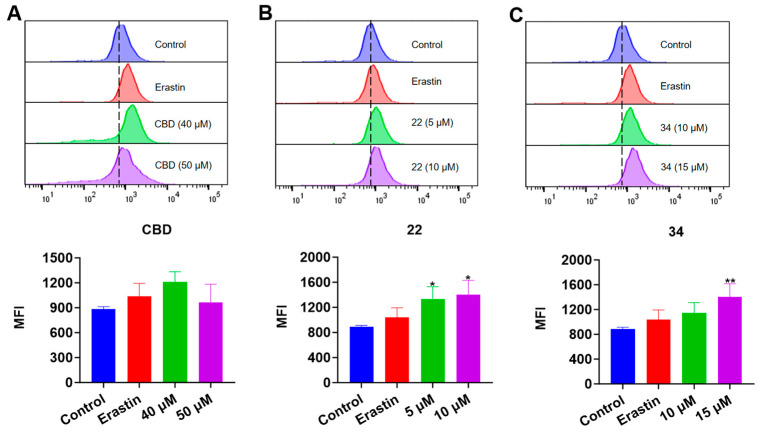
Evaluations of intracellular iron in B16F10 cells exposed to erastin, CBD, **22**, and **34**. Plots of a flow cytometry assay with the FerroOrange staining reagent showing the intracellular iron levels (upper panel; data are shown as the PE values) and quantitative analysis of the Fe^2+^ level (lower panel; data are expressed as the mean ± SD MFI values) in B16F10 cells exposed to CBD, **22**, and **34** (**A**–**C**). Significance was defined as * *p*  < 0.05 and ** *p* < 0.01 when compared to the control group.

In the FerroOrange assay, the iron accumulation in B16F10 cells exposed to erastin (15 μΜ), CBD, **22**, and **34** was detected. While the fluorescent signal of cells exposed to CBD was insignificant, treatment with compound **22** (5 and 10 μM) resulted in an elevation of fluorescence signals by 50.7% and 57.9%, respectively, compared to the control group. Similarly, treatment with compound **34** at 15 μM led to an increased fluorescence signal by 58.8% (Figure 9). These results showed that CBD and its derivatives (**22** and **34**) had a weak effect on the iron accumulation in B16F10 cells, which suggests that these compounds may not suppress the cell growth via ferroptosis given that the elevation of iron level in cells is a critical characteristic of ferroptosis.

### 3.6. CBD and Its Bipiperidinyl Derivatives Elevate Intracellular ROS in B16F10 Cells

**Figure 10:**
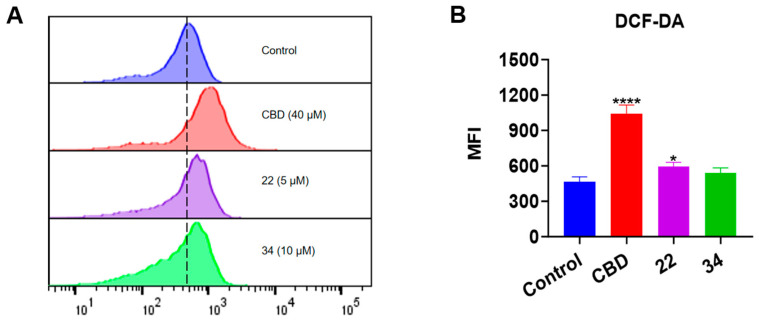
Intracellular ROS levels in B16F10 cells exposed to CBD, **22**, and **34**. (**A**) Flow cytometry analysis of intracellular ROS levels using the DCF-DA probe. (**B**) Quantitative analysis of intracellular ROS levels detected by the fluorescence signals. Data are shown as the averaged FITC and MFI values. Data are expressed as the mean ± SD values. Values are expressed in means ± SD from three experiment replicates. Significance was defined as * *p* < 0.05 and **** *p* < 0.0001 when compared to the control group.

Given that both apoptosis and ferroptosis can trigger cell death by increasing the production of intracellular reactive oxygen species (ROS) [21], we measured the effect of CBD and its derivatives on the production of ROS in B16F10 cells using the DCF-DA assay (Figure 10). CBD (40 μM) showed a promising effect in elevating the ROS level (by 1.23-fold) compared to the control group. Although compounds **22** and **34** both had a trend of promoting ROS production, only **22** (5 μM) significantly increased the level of cellular ROS by 27.2% (Figure 10B).

### 3.7. CBD and Its Bipiperidinyl Derivatives’ Effect on Pyroptosis in B16F10 Cells

**Figure 11:**
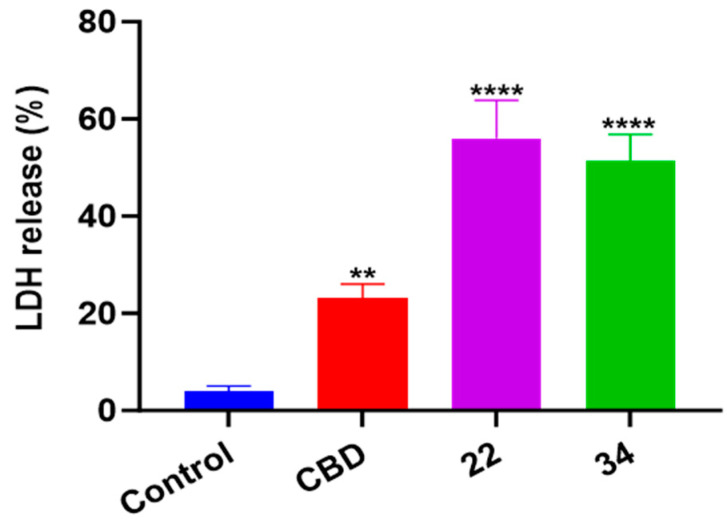
The level of cell supernatant dehydrogenase (LDH) in B16F10 cells treated with CBD, **22**, and **34**. The fluorescence intensity was measured by an LDH kit and values are expressed in means ± SD from three experiment replicates. Significance was defined as ** *p* < 0.01 and **** *p* < 0.0001 when compared to the control group.

In addition to oxidative stress, cellular inflammatory stress can also trigger a specific type of programmed cell death known as pyroptosis. In this study, the effects of CBD, **22,** and **34** on pyroptosis in B16F10 cells were evaluated by measuring the level of cell supernatant dehydrogenase (LDH), which is a biomarker for damage to cell membrane integrity. At the concentrations used in the apoptosis and ferroptosis assays, CBD, **22**, and **34** had no significant effects on the release of LDH nor cytokine (IL-1) in B16F10 cells. These compounds at a higher concentration (40 μM) increased the production of LDH by 19.0%, 52.0%, and 47.3%, respectively, compared to the control group (Figure 11). This suggests that pyroptosis was not a major contributor to the overall B16F10 cell death induced by CBD and compounds **22** and **34**.

It is reported that cannabinoids may alleviate tumor progression by initiating programmed cell death (e.g., apoptosis) to inhibit cancer cell proliferation. Cannabinoids confer the anti-tumor activity by the induction of apoptosis through cannabinoid receptors 1- and 2-dependent stimulation [22]. In particular, THC was reported to induce the apoptotic death of glioma cells via the production of pro-apoptotic sphingolipids [23]. Although this mechanism is less clear with CBD, studies proposed that CBD may promote cancer cells’ apoptotic death independently of CB1 and CB2 receptors [24]. Regardless of the involvement of cannabinoid receptors, it seems that the promotion of apoptotic cell population in cancer cells is due to cannabinoids’ capacity to stimulate ROS production [25]. This is in agreement with our observation that CBD increased the cellular ROS in B16F10 cells (Figure 10). In addition, studies showed that CBD can trigger the depletion of glutathione (GSH) to aggravate oxidative stress and cell death in human glioma cells [26]. This suggests that CBD’s effects on cellular redox homeostasis may play a critical role in its anti-proliferative effects in cancer cells. Given that GSH and ferroptosis are interconnected in the context of cellular biology, we evaluated the effects of CBD and its bipiperidine derivatives on the lipid peroxidation and cellular iron level (which are the characteristic biomarkers of ferroptosis) in B16F10 cells. Data from the Liperfluo and FerroOrange assays showed that CBD and its bipiperidine derivatives only alter lipid peroxidation but not iron accumulation in B16F10 cells. Thus, it is not clear whether these cannabinoids can effectively induce ferroptosis in melanoma cells. Further experiments using specific ferroptosis inhibitors (such as ferrostatin-1 [27]) are warranted to explore CBD’ and its bipiperidine derivatives’ potential role in ferroptosis in melanoma cells. Notably, our group reported that CBD can alleviate erastin-induced ferroptosis in noncancer skin cell lines (HaCaT cells) [28], suggesting that CBD’s effect on ferroptosis in skin normal cells and cancer cells is different. Moreover, our data showed that CBD and its bipiperidine derivatives had a weak effect on releasing LDH in B16F10 cells, which suggests that CBD and compounds **22** and **34** did not induce pyroptosis to exert an antiproliferative effect. Interestingly, our group reported that CBD can protect human skin keratinocytes from oxidation-induced pyroptosis by reducing cellular LDH levels [7]. This suggests that CBD may have selective effects on pyroptosis in different types of skin cells. A similar modulatory effect of CBD on pyroptosis in normal and cancer cells has been reported. CBD can suppress the growth of hepatocellular carcinoma cells by inducing a caspase-3/GSDME-dependent manner [29], whilst CBD showed an alleviative effect against alcohol-induced liver damage by regulating the NLRP3–pyroptosis pathway [30]. However, mechanistic studies are warranted to elucidate CBD’s role in mediating pyroptosis for skin protection. Nevertheless, this is the first study showing that the antiproliferative effect of CBD against melanoma cells can be improved by introducing a bipiperidine moiety. In addition, CBD and its bipiperidine derivatives suppress the growth of B16F10 cells by mediating programmed cell death (i.e., apoptosis), which provides useful information for developing cannabinoid-based anti-tumor agents.

## 4. Conclusions

In summary, a series of phytocannabinoids were evaluated for their antiproliferative effects against melanoma cells (B16F10 and A375) and CBD showed the most promising activity. In addition, chemical modifications by introducing a bipiperidinyl group in CBD resulted in a pair of CBD derivatives (**22** and **34**) with enhanced cytotoxicity on B16F10 and A375 cells. Furthermore, data from a panel of bioassays supported the notion that the enhanced antiproliferative effects of CBD and its bipiperidinyl derivatives were associated with their capacity to mediate programmed cell death such as apoptosis in B16F10 cells. Further studies on the anti-tumor effect of CBD and its bipiperidinyl derivatives with in vivo models are warranted to better understand their effectiveness in the potential development of melanoma management.

## Figures and Tables

**Table 1:** Effects of phytocannabinoids on the cell viability of murine (B16F10) and human (A375) melanoma cells.

Cell Viability (as of Control%)					
Compd (10 µM)	B16F10	A375	Compd (100 µM)	B16F10	A375
CBD	135.0 ± 2.0	100.8 ± 4.7	CBD	8.5 ± 0.3	5.3 ± 0.3
CBG	102.8 ± 2.6	84.8 ± 1.9	CBG	15.3 ± 1.2	7.0 ± 0.4
CBT	101.8 ± 5.0	84.5 ± 3.8	CBT	69.0 ± 2.0	76.3 ± 6.4
CBN	105.5 ± 2.9	97.8 ± 4.6	CBN	6.8 ± 0.5	6.5 ± 0.3
CBC	101.3 ± 0.7	93.0 ± 3.7	CBC	54.7 ± 7.5	47.7 ± 2.4
CBGA	95.7 ± 2.1	72.8 ± 6.4	CBGA	36.0 ± 2.1	65.5 ± 3.0
CBDA	92.3 ± 2.7	74.3 ± 4.3	CBDA	62.8 ± 2.1	75.3 ± 0.8
CBDV	99.0 ± 2.0	89.0 ± 2.9	CBDV	14.3 ± 2.0	17.3 ± 1.5
Δ8-THC	117.0 ± 1.7	103.8 ± 1.7	Δ8-THC	6.3 ± 0.3	6.8 ± 0.5

**Table 2:** The antiproliferation IC_50_ values of phytocannabinoids on B16F10 and A375 melanoma cells.

		IC_50_ (μM)			
Cell line	CBD	CBG	CBN	CBDV	Δ8-THC
B16F10	28.6	50.3	31.9	53.2	37.5
A375	51.6	56.0	64.0	51.3	50.9

**Table 3:** Inhibitory effects of two sets of CBD derivatives with various functional groups (**13**–**36**) on the growth of B16F10 and A375 cells at the concentration of 20 μM.

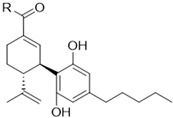					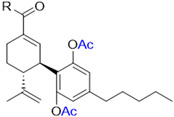			
Compound	Inhibition		R Group			Compound	Inhibition	
	B16F10	A375					B16F10	A375
**13**	n.a. ^a^	14.7%	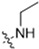			**25**	4.5%	17.7%
**14**	n.a.	67.0%	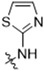			**26**	42.2%	47.0%
**15**	41.7%	55.7%	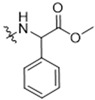			**27**	27.5%	21.2%
**16**	35.2%	69.5%	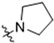			**28**	22.2%	11.1%
**17**	1.5%	34.7%	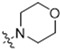			**29**	36.7%	13.4%
**18**	46.5%	31.2%	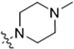			**30**	66.2%	46.2%
**19**	48.0%	38.5%	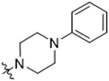			**31**	n.a.	48.5%
**20**	11.5%	12.7%	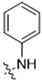			**32**	21.2%	49.5%
**21**	n.a.	6.2%	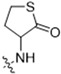			**33**	n.a.	22.2%
**22**	86.5%	89.1%	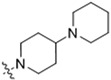			**34**	88.5%	76.5%
**23**	66.5%	17.3%	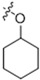	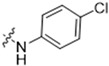		**35**	6.0%	11.2%
**24**	13.5%	12.7%	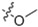	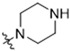		**36**	32.2%	51.1%

^a^ n.a. = not active (no inhibition).

## Data Availability

Raw data obtained in this study are available from the corresponding author upon reasonable request.

## Supplementary Materials

The following supporting information can be downloaded at https://www.mdpi.com/article/10.3390/antiox13040478/s1, Table S1: Inhibitory effects of the reaction intermediate **37**–**56** with a leaving group triflate (-OTf) or a protecting group pivaloyl (-Piv) on the growth of B16F10 cells at the concentration of 20 μM; Table S2: Inhibitory effects of the synthetic cannabinoids **50**–**56** with various side chains on the growth of B16F10 cells at the concentration of 20 μM.
