# Supplementary material for: Bipiperidinyl Derivatives of Cannabidiol Enhance Its Antiproliferative Effects in Melanoma Cells
Source: Antioxidants (Basel). 2024 Apr 17;13(4):478. doi: 10.3390/antiox13040478 (PMC11047683; doi:10.3390/antiox13040478)
Supplement: Supplementary file 1 [file antioxidants-13-00478-s001.zip › antioxidants-2914694-supplementary.pdf]

SUPPLEMENTARY MATERIALS

Bipiperidinyl Derivatives of Cannabidiol Enhance Its Antiproliferative Effects in Melanoma Cells

Peihong Lyu [1, 2], Huifang Li [1], Junzhao Wan [3], Ying Chen [1, 4], Zhen Zhang [5], Panpan Wu [5], Yinsheng Wan [6], Navindra P. Seeram [1], Jean Christopher Chamcheu [7, 8], Chang Liu [1], and Hang Ma [1*]

[1] Bioactive Botanical Research Laboratory, Department of Biomedical and Pharmaceutical Sciences, College of Pharmacy, University of Rhode Island, Kingston, RI 02881, USA
[2] Department of Dermatology, Affiliated Hospital of Guizhou Medical University, Guiyang, Guizhou 550001, China
[3] School of Pharmacy, Guizhou Medical University, Guiyang, Guizhou 550001, China.
[4] Department of Obstetrics and Gynecology, The Second Affiliated Hospital of Soochow University, Suzhou 215004, China
[5] School of Pharmacy and Food Engineering, Guangdong Provincial Key Laboratory of Large Animal Models for Biomedicine, Wuyi University, Jiangmen 529020, China
[6] Department of Biology, Providence College, Providence, RI 02918, USA
[7] Department of Biological Sciences and Chemistry, College of Sciences and Engineering, Southern University and A&M College, Baton Rouge, LA 70807, USA
[8] Department of Pathobiological Sciences, School of Veterinary Medicine, Louisiana State University, Baton Rouge, LA 70803, USA

Table S1. Inhibitory effects of the reaction intermediate **37-49** with a leaving group triflate (-OTf) or a protecting group pivaloyl (-Piv) on the growth of B16F10 cells at the concentration of 20 μM.

Compound	MW (g/mol)	Functional group	Inhibition
**37**	260.33	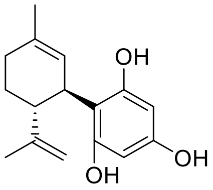	n.a.[a]
**38**	392.39	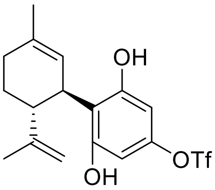	n.a.
**39**	560.63	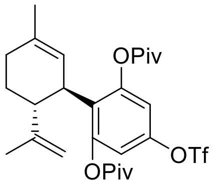	n.a.

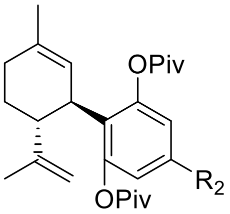

Compound	MW(g/mol)		Functional group	Inhibition
**40**	426.60	R_2=	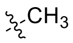	n.a.
**41**	440.62	R_2=	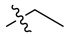	n.a.
**42**	452.64	R_2=	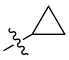	n.a.
**43**	454.65	R_2=	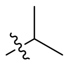	n.a.

**44**	494.72	R2=	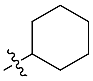	n.a.
**45**	480.69	R2=	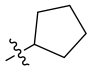	n.a.
**46**	488.67	R2=	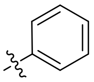	n.a.

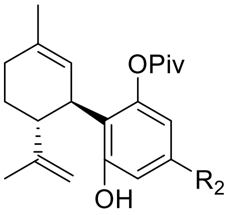

Compound	MW(g/mol)		Functional group	Inhibition
**47**	370.53	R2=	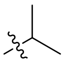	n.a.
**48**	410.60	R2=	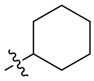	32.3%
**49**	396.57	R2=	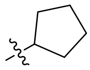	49.0%

[a]n.a. = not active (no inhibition)

Table S2. Inhibitory effects of the synthetic cannabinoids **50-56** with various side chains on the growth of B16F10 cells at the concentration of 20 μM.

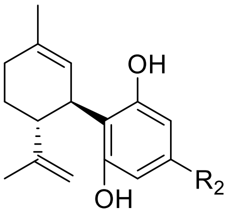

Compound	MW(g/mol)		Functional group	Inhibition
**50**	258.36	R2=	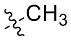	n.a.[a]
**51**	272.39	R2=	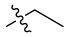	n.a.
**52**	284.40	R2=	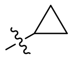	n.a.
**53**	286.42	R2=	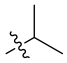	n.a.
**54**	326.48	R2=	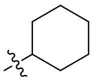	61.7%
**55**	312.45	R2=	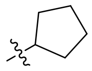	55.2%
**56**	320.43	R2=	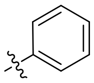	n.a.

[a]n.a. = not active (no inhibition)
